# 3D cellular-resolution imaging in arteries using few-mode interferometry

**DOI:** 10.1038/s41377-019-0211-5

**Published:** 2019-11-21

**Authors:** Biwei Yin, Zhonglie Piao, Kensuke Nishimiya, Chulho Hyun, Joseph A. Gardecki, Adam Mauskapf, Farouc A. Jaffer, Guillermo J. Tearney

**Affiliations:** 1 Wellman Center for Photomedicine, Harvard Medical School and Massachusetts General Hospital, Boston, MA 02114 USA; 2 Cardiovascular Research Center and Cardiology Division, Harvard Medical School and Massachusetts General Hospital, Boston, MA 02114 USA; 3 Department of Pathology, Harvard Medical School and Massachusetts General Hospital, Boston, MA 02114 USA; 4 Harvard-MIT Division of Health Sciences and Technology, Cambridge, MA 02139 USA

**Keywords:** Biophotonics, Imaging and sensing, Fibre optics and optical communications

## Abstract

Cross-sectional visualisation of the cellular and subcellular structures of human atherosclerosis in vivo is significant, as this disease is fundamentally caused by abnormal processes that occur at this scale in a depth-dependent manner. However, due to the inherent resolution-depth of focus tradeoff of conventional focusing optics, today’s highest-resolution intravascular imaging technique, namely, optical coherence tomography (OCT), is unable to provide cross-sectional images at this resolution through a coronary catheter. Here, we introduce an intravascular imaging system and catheter based on few-mode interferometry, which overcomes the depth of focus limitation of conventional high-numerical-aperture objectives and enables three-dimensional cellular-resolution intravascular imaging in vivo by a submillimetre diameter, flexible catheter. Images of diseased cadaver human coronary arteries and living rabbit arteries were acquired with this device, showing clearly resolved cellular and subcellular structures within the artery wall, such as individual crystals, smooth muscle cells, and inflammatory cells. The capability of this technology to enable cellular-resolution, cross-sectional intravascular imaging will make it possible to study and diagnose human coronary disease with much greater precision in the future.

## Imaging: A better resolution for diagnosing of coronary artery disease

A technique that provides subcellular-level images of the heart’s vascular system could allow scientists to study and diagnose human coronary disease with much greater precision. Intravascular optical coherence tomography is the conventional method for assessing the build-up of coronary plaque, which narrows arteries and leads to coronary artery disease. However, the technique has a lateral resolution of only 30 microns and is unable to provide images at the subcellular level, which is key to understanding the disease. Now, Guillermo Tearney and colleagues from Department of Pathology and Wellman Center at Massachusetts General Hospital and Harvard Medical School, in the United States, have developed a few-mode interferometry intravascular imaging system with a resolution of around 3 microns, and can provide images of cellular and subcellular structures within the artery wall, such as individual crystals, smooth muscle cells, and inflammatory cells.

## Introduction

Optical coherence tomography (OCT)^1^ is a mainstream cross-sectional, reflectance imaging technique that has found clinical utility for imaging a diverse range of human tissues, including those of the luminal organs inside the body^2^. Of particular interest is intravascular OCT (IVOCT), which is currently clinically available globally for accessing coronary plaque structure and guiding percutaneous coronary intervention (PCI) of coronary artery disease^3^, the number one cause of mortality in the world. More recently, advanced IVOCT technologies have been demonstrated, such as multimodal IVOCT that combines conventional IVOCT with other imaging or sensing modalities, such as fluorescence and near-infra-red spectroscopy^4–6^, polarisation-sensitive IVOCT that measures tissue birefringence to provide additional imaging contrast^7^, and heartbeat IVOCT that makes it possible to densely image coronary arteries in vivo without motion artefacts^8^.

Owing to the divergence of focused beams and the requirement of a large focal depth to maintain cross-sectional imaging capability, conventional OCT systems and probes acquire images with a lateral resolution of approximately 30 μm, relegating this technology to the assessment of architectural as opposed to cellular morphology. Since individual cells and subcellular structures are at the very foundation of the pathobiology of human disease^9^, substantial efforts have been made to increase the lateral resolution of OCT probes by at least an order of magnitude so that features at this scale can be visualised. This technical feat has been a daunting task and heretofore has not been realised in small-diameter, flexible probes in a practical and reliable manner.

The most challenging barrier to increasing the lateral resolution of an OCT system is that the depth of focus (DOF, commonly defined as twice the Rayleigh range) of the objective is proportional to the square of the focused spot size, which prohibits cross-sectional imaging when the beam waist is too small. Though studies have been reported for OCT probes with increased DOF^10–14^, they either have a DOF gain (approximately two- to threefold) that is not sufficient to account for variable luminal surface topology and uncontrollable probe–lumen distances^10–12,14^ or have a form factor or complexity that is not suitable for use in small-diameter, flexible probes^13^, making them ineffective for intraluminal clinical applications such as coronary imaging. Previously, we demonstrated that the self-imaging effect of multimode fibres can be applied for extended DOF coherent imaging^15^. With further exploration of the unique propagation properties of coaxially focused multimode beams and their applications for in vivo imaging, here, we describe a few-mode interferometry-based intravascular imaging system with a greatly extended DOF that allows cross-sectional imaging at cellular resolution (~3 μm) over a depth range of more than 1 mm. This technology enables the visualisation of cellular and subcellular structures of intact human-coronary-sized arteries ex vivo and in vivo through a submillimetre diameter, flexible catheter.

## Results

**Fig. 1:**
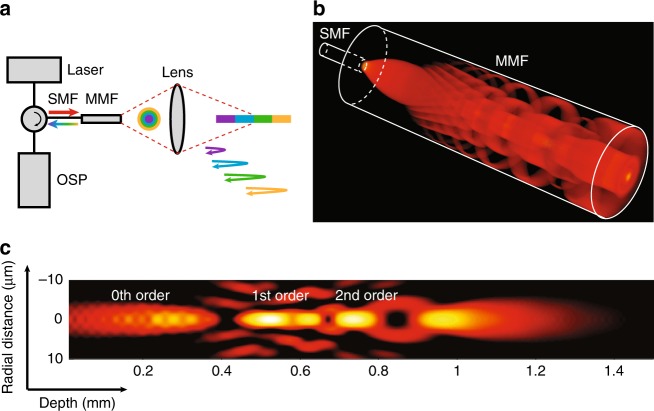
**a** The concept of using a single-mode-multimode fibre system for depth-encoded reflectometry. The colour pattern corresponds to different propagation modes. SMF single-mode fibre, MMF multimode fibre, OSP optical signal processing unit. **b** 3D rendering of the intensity profile inside the multimode fibre showing the few-mode generation processes of an SMM fibre system. The lateral and axial dimensions are not drawn to scale. The SMF has a mode field diameter of 5 μm, and the MMF has a core diameter of 50 μm and a length of 1.2 mm. A system wavelength of 800 nm is assumed. **c** Simulations of the focused field intensity distribution in the image space. The length of the spacer is 1.6 mm, and the objective has a focal distance of approximately 0.5 mm. We assume a refractive index of 1.34 in the image space. The focused field intensity distribution is normalised by the peak intensity and displayed on the dB scale with a dynamic range of 16 dB.

The few-mode interferometry imaging system is proposed for extended DOF imaging, as shown in Fig. 1a. A segment of a multimode fibre is spliced, with a single-mode fibre as a single-mode-multimode (SMM) fibre element, which generates LP_0*m*_-like modes^16^, where the mode index *m* corresponds to the number of circular propagation modes supported by the multimode fibre (Fig. 1b). In a conventional confocal imaging system, the spherical wavefront of a single diverging beam is modified by the phase function of an objective, converging onto one single focus. In contrast, for the SMM fibre system, due to the self-imaging property of multimode fibres, circular propagation modes with different angular spectra divide the wavefront of the beam into multiple annular zones. The field distribution *U*_*l*_ on the objective can be approximated as

1 $${\it{U}}_{\it{l}}\;\left( {{\it{r}}_{\it{l}}} \right) = {\it{P}}_{\mathrm{0}}\;\left( {{\it{r}}_{\it{l}}} \right){\it{G}}\left( {{\it{r}}_{\it{l}}} \right) + \mathop {\sum}\limits_{m = 1}^{\it{M}} {{\it{P}}_{\it{m}}\left( {{\it{r}}_{\it{l}}} \right){\it{G}}\left( {{\it{r}}_{\it{l}} + {\it{md}}} \right)}$$

where *r*_*l*_ is the radial coordinate in the objective plane, *d* is the core diameter of the multimode fibre, and *M* represents the number of circular modes. *G* represents a Gaussian beam function generated by the single-mode fibre, *P*_0_ is the circular aperture function for the 0th-order mode, and *P*_*m*_ is the annular aperture function for high-order modes (see Supplementary Section 1). The first term on the right-hand side of Eq. (1) represents the field corresponding to the 0th-order mode, which approximates Gaussian beam propagation, and the second term represents a coherent summation of the fields generated by the high-order modes. Due to the self-imaging effect, each high-order mode can be treated as a Gaussian beam identical to the 0th-order mode, but with a beam waist shifted by a distance *md* in the radial direction in response to *m*th-order reflection. The aperture function for the 0th-order mode (*P*_0_) is approximated as the projection of the multimode fibre emitting aperture onto the objective plane. Similarly, the annular aperture function for each high-order mode (*P*_*m*_) is approximated as the multimode fibre emitting aperture projected along the beam propagation path of each mode. After being focused by an objective, due to the difference in wavefront curvature, the high-order modes converge onto an axial region that is different from that of the 0th-order mode, and when all modes are added, an extended focusing region is created. Once the sample is illuminated by the beam, depth-resolved information of the sample encoded in all the modes is reflected and transformed by the objective onto a transverse plane and coupled back into the SMM fibre system, then transmitted to the optical signal processing unit through a single-mode fibre. Since the information carried by each mode travels a different optical pathlength, a low coherence interferometry that resolves pathlength delay is used for decoding. Thus, multiple propagation modes can simultaneously interrogate a sample at different depths, and the depth-encoded signal can be transmitted back through a common channel for processing, which essentially increases the acquisition capacity of a reflectometry system without additional illumination and detection channels.

**Fig. 2:**
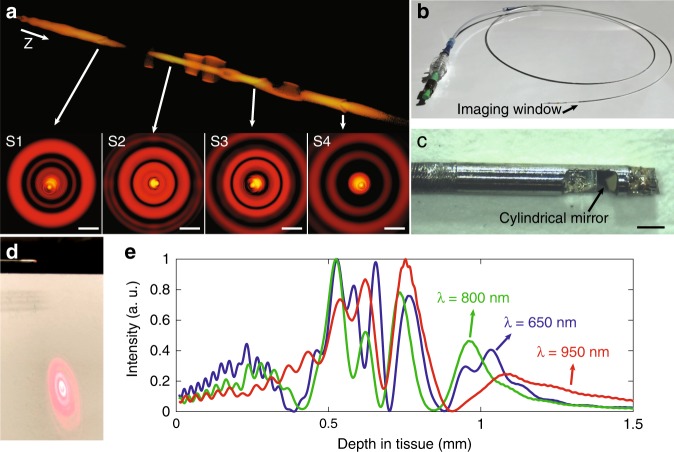
**a** Simulation of the aberrated beam field intensity when scatterers are positioned at the centre of the beam path. Four scatterers were modelled, denoted as S1–S4. The scatterers had a diameter of 2 μm and a refractive index of 1.5. 3D rendering of the beam field and the transverse intensity distributions show that the aberration introduced by an individual scatterer is confined within each mode. Z indicates the beam propagation direction. Scale bar: 10 μm. **b** Photograph of the completed 2.6-F rapid guidewire exchange coronary catheter. **c** A photograph of the distal end of the catheter, taken using a microscope. The fibre probe had a diameter of 500 μm and a rigid length of less than 4 mm. Scale bar: 500 μm. **d** Photograph of the ring pattern of the light transmitted through the catheter’s fibre probe optics, corresponding to multiple propagation modes. The screen was positioned at a small angle with respect to the beam propagation direction, showing that the cylindrical mirror directs the beam at an ~8° angle normal to the sheath to reduce specular reflection. **e** Simulation of the normalised on-axis field intensity distribution with respect to depth for the centre wavelength and the two ends of the spectra, showing that the chromatic focal shift effect mitigated the field intensity discontinuity.

The field intensity distribution in the image space is simulated by the beam propagation method^17^, as shown in Fig. 1c, where we observe a substantially elongated focusing region. In addition to the self-healing effect of the Bessel/pseudo-Bessel fields^18^ of the high-order modes, we found an additional mode-related self-healing effect in the overall focused beam field, which we call modal self-healing, as a unique propagation property of the SMM fibre system. To visualise this effect, we simulated the focused beam field in the image space through four 2-μm-diameter scatterers (refractive index of 1.5) at different depths along the centre of the beam path. In contrast to the original beam profile (Fig. 1c), these scattering particles introduce aberration to the beam field (Fig. 2a), shown as the field disturbance at the centre of the transverse beam profile. However, the effect of each scatterer is primarily confined to only the mode that is focused on the scatterer and does not substantially affect the other non-focusing modes. This effect was observed as the higher-order modes that correspond to the ring pattern on the transverse beam profile circumvent the scatterers, propagate with minimal disturbance, and converge onto deeper regions. This modal self-healing phenomenon suggests the propagation independence of each mode in the scattering media.

Based on the concept described above (Fig. 1a), we created an intravascular few-mode interferometry (IVFMI) imaging device consisting of a supercontinuum laser as the light source, a low-coherence interferometer as the optical signal processing unit, a catheter for depth encoding and backscattering signal detection, and optomechanics for scanning (Supplementary Section 2). The supercontinuum-source-based low-coherence interferometer measured the pathlength delay with a resolution of approximately 1.5 μm in tissue, and the sensitivity was approximately 92 dB at a 35-kHz A-line (depth-scan) rate with a 20-mW power incident on the sample. The clinical catheter (Fig. 2b), with an outer diameter of 870 μm, contained an SMM fibre element, a spacer, and a graded index (GRIN) objective lens. Light transmitted from the lens impacted a cylindrical mirror to direct the beam for side-view imaging; the mirror compensated astigmatism caused by the enclosing catheter’s sheath. Using the design shown in Fig. 1b, c, approximately three modes were excited by the SMM fibre element. The reflection from the GRIN lens’ distal end surface was used as the interferometer's reference signal. The fibre probe was protected by a drive shaft (Fig. 2c), which resided within the catheter’s outer sheath. The in-house fabricated cylindrical mirror^19^ was tilted so that the light was at an 8° angle normal to the sheath to reduce specular reflection (Fig. 2d). It is important to note that in addition to the SMM fibre element, this catheter was identical to coronary catheters that we and others have used for IVOCT imaging in living human patients^20^. The proximal end of the catheter was connected to a custom-built broadband optical rotary junction (MJP, Princetel Inc., NJ) mounted on a linear translation stage. A helical scan of the lumen wall was performed, enabling 3D reconstruction of the artery by simultaneously spinning (17 Hz) and axially translating the catheter’s fibre probe and driveshaft (10–200 μm/frame). When the catheter was inside an artery, cross-sectional images comprising 2048 A-lines were acquired at 17 frames/s. Since a broad-bandwidth source was used, we simulated the SMM fibre system output at the centre wavelength (800 nm) and two ends of the spectra (650 and 950 nm). The field intensity discontinuity caused by mode transition manifested as the intensity fall-off was mitigated by the chromatic focal shift effect, as shown in Fig. 2e. However, due to the modification of the spectrum at the mode transition zones, the axial resolution at these depths may be reduced.

The characterisation results demonstrated that the catheter had an average lateral resolution of 3–4 μm, starting from the outer surface of the sheath and extending to 1.5 mm away from the centre of the catheter; the axial resolution of the catheter was maintained at an average of 1.5 μm in tissue. The significantly extended DOF increased the depth-imaging capabilities of the catheter by more than one order of magnitude. When compared with a Gaussian beam that has a beam waist with a full-width-at-half-maximum of 3 μm in a medium with a refractive index of 1.5, the phantom image demonstrated that the catheter resolution can be maintained over a 1-mm range, corresponding to a 13-fold improvement, making it now possible to obtain cross-sectional, cellular-resolution intravascular images of a 3-mm-diameter circular cylinder when spinning. Note that the DOF characterised by the phantom image was shorter than the theoretical prediction due to the scattering attenuation and system sensitivity roll-off.

**Fig. 3:**
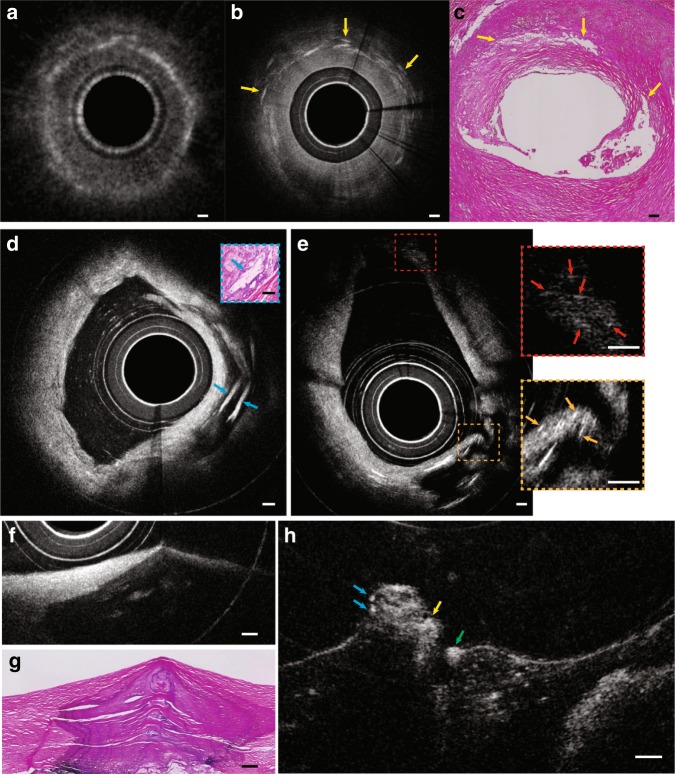
**a**–**c** IVOCT, IVFMI, and histology images showing a cross-section of the artery containing deposits of cholesterol crystals. In the standard IVOCT image (**a**), the highly scattering structures would be considered macrophage accumulations using current IVOCT criteria^21^, while the IVFMI image (**b**) demonstrates that these features were crystals, a finding that is consistent with the corresponding histology. **d** A cross-section of an artery that had multiple cholesterol crystals characterised by reflections from their top and bottom surfaces. **e** Image showing that IVFMI could resolve small crystals at distances close to the sheath (a couple hundred microns) and far from the sheath (~1 mm) simultaneously. **f**, **g** IVFMI and corresponding histology images of a calcific nodule, respectively. **h** was approximately 1.3 mm away longitudinally from (**g**), where thrombus was observed over the calcific nodule. The blue arrows are features that are consistent with leucocytes, the yellow arrow is suggestive of thrombus, and the green arrow shows a cell that is likely a monocyte/macrophage. A Gaussian blur filter with a radius of 2 μm was applied to the cross-sectional IVFMI images. Scale bars for all images are 100 μm.

**Fig. 4:**
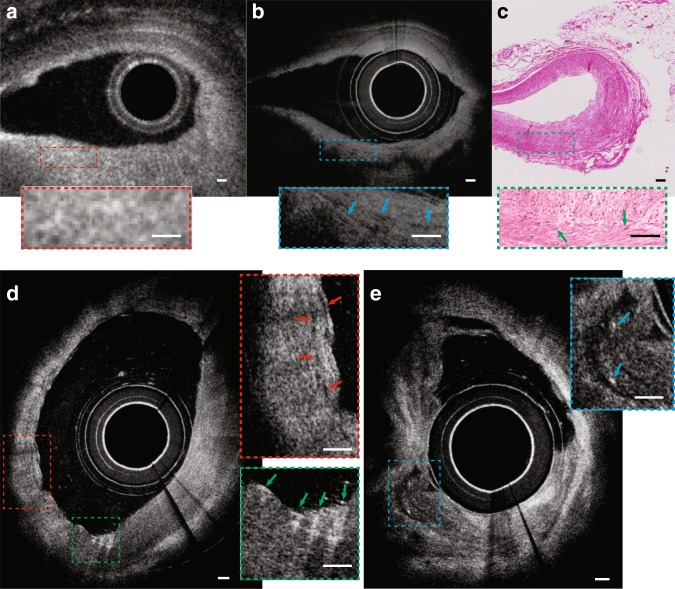
**a**–**c** IVOCT, IVFMI, and histology images showing a cross-section of the cadaveric coronary artery with smooth muscle cells residing within a collagen network. The smooth muscle cells could not be clearly visualised in the standard IVOCT image, while IVFMI showed smooth muscle cells (low-intensity, slit-like structures indicated by blue arrows in the inset of (**b**)) that are consistent in size and shape with the smooth muscle cells seen in the corresponding histology (green arrow in the inset of (**c**)). **d** IVFMI image of a cross-section of an artery that shows probable smooth muscle cells (red arrows in the inset) and macrophages undergoing diapedesis (green arrows in the inset). **e** IVFMI image of a cross-section of a cadaver coronary artery showing probable thrombus, with cells that are consistent with leucocytes embedded in the intraluminal mass. A Gaussian blur filter with a radius of 2 μm was applied to the cross-sectional IVFMI images. Scale bars are 100 μm for all images.

With an almost 1000-fold improvement in volumetric resolution, cellular and subcellular structures that could not be resolved by conventional IVOCT could now be visualised by IVFMI. Standard IVOCT (with an axial resolution of ~10 μm and a lateral resolution of ~30 μm) and IVFMI images corresponding to the same cross-section of a human cadaver coronary artery are presented for comparison in Fig. 3a, b and Fig. 4a, b. Densely packed crystals located a few hundred microns below the luminal surface could be clearly distinguished only with IVFMI (Fig. 3b). With standard IVOCT (Fig. 3a), they were blurred and globular, making them more likely to be characterised as macrophage accumulations, according to current IVOCT image interpretation criteria^21^. Similarly, individual smooth muscle cells were observed by the IVFMI catheter (Fig. 4b) and not resolved by conventional IVOCT (Fig. 4a).

Figure 3d shows an IVFMI cross-section of an artery that contains cholesterol crystals that were notable for reflections seen at both the top and bottom of the crystals, a feature that could not be resolved by conventional IVOCT. Figure 3e presents another cross-section of a coronary artery that demonstrates crystals residing at different locations within the plaque. Owing to the extended DOF, these microstructures residing at a distance of a couple hundred microns to approximately a millimetre away from the catheter sheath were simultaneously resolved by IVFMI in one circumferential scan. Figure 3f, g shows IVFMI images and a corresponding histology of a calcific nodule, one of the plaque types associated with an increased risk for precipitating a coronary event^22^, with an overlying fibrous cap. Figure 3h is IVFMI of the same calcific nodule at a different longitudinal location; a thrombus formed on the portion of the calcific nodule that juts out into the lumen; cells consistent with leucocytes were seen adhering to the nodule; and a cell likely to be a macrophage was evident to the right of the nodule.

Inflammatory cells are at the very heart of the development of atherosclerotic plaques and their clinical sequelae. Figure 4d is a cross-sectional image obtained with the IVFMI that may represent intimal smooth muscle cells and macrophages undergoing diapedesis. Figure 4e also shows what appears to be thrombus, demonstrating fine details in the intraluminal mass, including bright cells that are likely to be leucocytes embedded in the fibrin mesh.

**Fig. 5:**
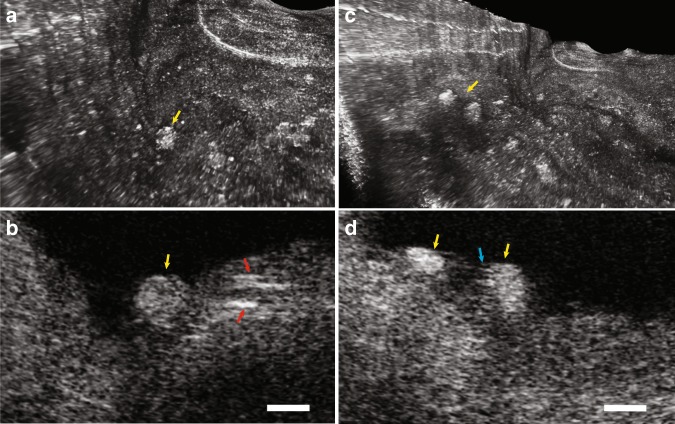
3D reconstruction and corresponding cross-sectional images of IVFMI data obtained from a human cadaver coronary artery. The lumen shows individual macrophages residing on the surface of a fibroatheromatous plaque. **a**, **b** 3D rendering and cross-sectional images showing an individual cell (yellow arrows) that appears to be transmigrating through the endothelium towards a deposit of intimal crystals (red arrows). **c**, **d** 3D rendering and cross-sectional images showing two macrophages tethered to the endothelial surface, polarised towards one another with extended pseudopodia (blue arrow). Scale bars: 50 μm.

Figure 5a, c are 3D reconstructions of IVFMI data obtained from a portion of a cadaver coronary lumen wall showing macrophages adhering to the endothelium. Individual macrophages on the luminal surface were observed adjacent to intimal crystals (Fig. 5a, b). In another location, the IVFMI image shows structures that could be two macrophages with touching pseudopodia in communication with each other (Fig. 5c, d).

**Fig. 6:**
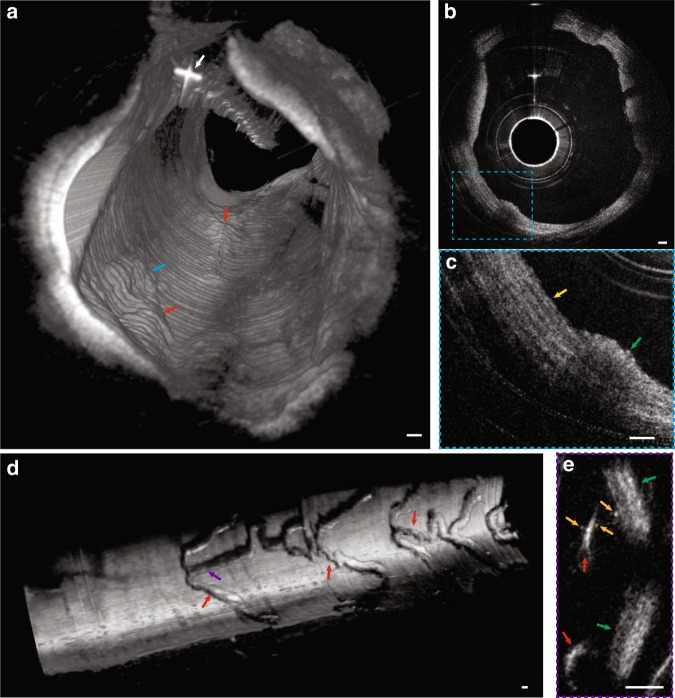
IVFMI images of rabbit arteries acquired in vivo. **a** 3D reconstruction of atherosclerotic rabbit aorta. The white arrow indicates the guide wire. The blue and red arrows indicate atherosclerotic plaque regions. **b** Cross-sectional image of the lumen wall that contains normal aortic media tissue and the atheromatous lesion indicated by the blue arrow in (**a**). **c** Magnified image corresponding to the blue dashed region in (**b**). The yellow arrow demarcates a region containing smooth muscle cells embedded in a collagen network in a portion of the normal aortic wall, while the green arrow indicates an atherosclerotic plaque. **d** 3D reconstruction of a stent implanted in the iliac artery. The purple and red arrows indicate stent struts. **e** A cross-sectional image corresponding to the location indicated by the purple arrow in (**d**). Orange arrows highlight tiny, punctate, highly scattering features that are consistent with platelets around the stent strut (red arrow), with the green arrows indicating the artery wall. A Gaussian blur filter with a radius of 2 μm was applied to the cross-sectional IVFMI images. Scale bar: 100 μm.

Figure 6a is a 3D reconstruction of IVFMI data obtained from a living rabbit aorta with atherosclerotic plaque. Plaques could be clearly discerned from the normal artery wall by their raised surface morphology that projected into the lumen. In cross-sectional images (Fig. 6b, c), a network of collagen and smooth muscle cells could be seen in the normal media, features that were disorganised and less visible in the intimal atherosclerotic lesion. Figure 6d is a 3D reconstruction of IVFMI data obtained in vivo at a segment of the lumen wall that was implanted with a stent 1 h before imaging. The capacity of IVFMI to enable the visualisation of the microstructural detail of the struts (Fig. 6d, red arrows) is unprecedented for intravascular imaging. Small, high-reflectivity, micron-sized dots were observed around some of the stent struts (Fig. 6e, orange arrows). The size and location of these features suggest that these microstructures were platelets.

## Discussion

Cellular-resolution, cross-sectional imaging using small-diameter catheters has been elusive in the field owing to the narrow usable imaging range when the beam emanating from the optical fibre is focused onto a small spot. This issue is further problematic when imaging luminal organs inside the body, where the device is at an unknown distance from the lumen wall and the luminal surface is irregular. In this study, we demonstrated a technique that overcomes these problems by implementing few-mode interferometry that increases the DOF by more than an order of magnitude. The novel mode division multiplexing/demultiplexing method enables the use of multiple time-delayed circular modes for imaging signal encoding and decoding in a single-mode-multimode fibre system. Because of its small footprint, depth-encoding capability, and transmission stability, this optical configuration has important applications in depth-resolved endomicroscopy. A clinically adaptable system based on few-mode interferometry for intravascular imaging was developed. The multiple propagation modes with encoding image information transmitted in parallel substantially increase the system acquisition capacity without adding an extra physical data path. Though the pseudo-Bessel beam fields corresponding to the high-order modes could present reduced collection efficiency and produce side-lobe artefacts compared to the conventional Gaussian beams^23^, the individual modes used in this technology capture a significant fraction of the reflected spatial frequencies. Thus, our results confirm that this technology is capable of acquiring images with a good signal-to-noise ratio showing well-defined, disease-relevant cellular and subcellular microstructures in human cadaver coronary arteries ex vivo and coronary-sized rabbit arteries in vivo. IVFMI images demonstrated that a diverse range of cells relevant to CAD can be visualised, including individual leucocytes, smooth muscle cells, and macrophages adherent to and transmigrating through the endothelium. Important extracellular microstructures were also observed, including cholesterol crystals and individual platelets. Since our device is physically and mechanically identical to coronary catheters that have been used for conventional IVOCT imaging in patients^20^, these findings indicate that we now have the capability to visualise cellular coronary pathology in humans in the cardiac catheterisation laboratory. Beyond intravascular imaging, the technology presented here will enable cellular imaging of other luminal organs, such as the gastrointestinal tract^24,25^ and pulmonary tracts^26^, potentially increasing diagnostic accuracy for other important diseases such as cancer.

## Materials and methods

### Resolution characterisation

Lateral and axial resolutions were measured by using the finished catheter to image a nanoparticle phantom (point spread function (PSF) phantom, National Physical Laboratory, UK) that contains iron oxide particles with a diameter of less than 1 μm suspended in an optically clear polyurethane resin with a refractive index of ~1.48 at 800 nm. PSFs were measured according to the intensity distribution of the nanoparticles imaged at various depths. Images were transformed to Cartesian coordinates prior to measurement. A cross-sectional image of the nanoparticle phantom was obtained with the catheter for characterisation of the PSF in the axial and lateral directions (Supplementary Section 3).

### Cadaver human coronary imaging ex vivo

Freshly excised diseased cadaver human coronaries were procured. The three main epicardial coronaries (left anterior descending coronary artery, right coronary artery, and left circumflex coronary artery) were prosected from the heart, warmed to 37 °C, placed in phosphate-buffered saline (PBS), flushed with PBS at a pressure of 100 mm Hg, and imaged by both IVFMI and conventional IVOCT. Once imaging was finished, the arteries were pressure-perfused with 10% buffered formalin at 100 mm Hg for 1 h to preserve their shape. Arteries were fixed for longer times (~48 h) and then underwent standard H&E histological processing. This study was approved by MGH IRB #2004P000578.

### Rabbit arteries imaging in vivo

Rabbit arteries with arterial-disease-relevant features were imaged by IVFMI in vivo. Two models were used for the study: (1) Atherosclerosis-bearing New Zealand white rabbits that underwent a 2-week high cholesterol diet (HCD) and abdominal aortic balloon injury, followed by a 4-week HCD and a subsequent 4-week normal chow regimen^27^; (2) New Zealand white rabbits with bare metal stents implanted in the iliac (multi-link mini vision coronary stent system 2.5 × 12 mm, Abbott Vascular, USA) 1 h before imaging. The rabbits were anaesthetised prior to imaging. The intravascular catheter was advanced to the aorta or iliac artery over a guide wire. During the administration of a contrast flush, the catheter helically scanned the arteries to acquire 3D datasets. All rabbit studies were approved by MGH IACUC #2013N000015.

## Supplementary information

supplementary information
