# Supplementary material for: 3D cellular-resolution imaging in arteries using few-mode interferometry
Source: Light Sci Appl. 2019 Nov 21;8:104. doi: 10.1038/s41377-019-0211-5 (PMC6872567; doi:10.1038/s41377-019-0211-5)
Supplement: Supplementary file 1 — supplementary information [file 41377_2019_211_MOESM1_ESM.docx]

**Supplementary information**

# **3D cellular-resolution imaging in arteries using few-mode interferometry**

Biwei Yin,^1^ Zhonglie Piao,^1^ Kensuke Nishimiya,^1^ Chulho Hyun,^1^ Joseph A. Gardecki,^1^ Adam Mauskapf,^2^ Farouc A. Jaffer,^1,2^ and Guillermo J. Tearney^1,3,4^

^1^Wellman Center for Photomedicine, Harvard Medical School and Massachusetts General Hospital, Boston, MA, USA 02114

^2^Cardiovascular Research Center and Cardiology Division, Harvard Medical School and Massachusetts General Hospital, Boston, MA, USA 02114

^3^Department of Pathology, Harvard Medical School and Massachusetts General Hospital, Boston, MA, USA 02114

^4^Harvard-MIT Division of Health Sciences and Technology, Cambridge, MA, USA 02114

1. **Self-imaging wavefront division**

In Eq. 1, *G* represents a Gaussian beam function generated by the single-mode fiber, and when the propagation distance is much longer than its Rayleigh range, it can be approximated as:

 (S1)

where *A* is the axial amplitude of the Gaussian beam propagating over a distance of *L*+*s* in a medium with a refractive index of *n*. *L* and *s* correspond to the length of the multimode fiber and the distance from multimode fiber to the objective, respectively. *w* is the radius of the 1/*e* amplitude, and *k* is the wave number.

*P*_0_ is the circular aperture function for the 0^th^ order mode, written as:

 (S2)

*P_m_* is the annular aperture function for high order modes, written as:

 (S3)

1. **Intravascular few-mode interferometry (IVFMI) imaging system**

A supercontinuum laser (SuperK Extreme EXR-15, NKT Photonics, Denmark) was employed to deliver broadband light onto sample through an intracoronary catheter. The backscattered light from the sample was interfered with light reflected by an optical surface at the catheter’s distal end (common-path interferometer) and detected by a broad-bandwidth high-spectral-resolution spectrometer (Fig. S1A). A depth-resolved reflectivity profile (A-scan) of the sample was recovered by Fourier-transforming the interference signal spectrum. The imaging system with a spectral detection window of 650-950 nm enabled acquisition of images with an axial resolution of 1.5 μm in tissue, and the 6-dB sensitivity roll-off was at a distance close to 1.5 mm. The catheter’s fiber probe incorporated a single-mode-multimode (SMM) fiber element that introduced multiple propagation modes that were coaxially focused onto the sample for extended depth of focus (DOF) imaging. As shown in Fig. S1B, the fiber probe consisted of a single-mode fiber (630HP, Nufern, CT) that transmitted light, a multimode fiber (FG050UGA, Thorlabs Inc., NJ) with a length of 1.2 mm that split the beam into approximately 3 propagation modes covering different spatial frequency bands of the wavefront, a spacer with a length of 1.6 mm that expanded the beam, a graded-index (GRIN) lens (GT-LFRL-050, Grintech GmbH, Germany) that focused the beam onto sample, and a cylindrical mirror that directed the beam toward the lumen wall and compensated astigmatism induced by the catheter’s 870-μm-diameter (2.6F) outer sheath. As the system has an axial resolution of 1.5 μm and a lateral resolution of 4 μm approximately, we expect each coherent volume to be of a size of 1.5 μm X 4 μm X 4 μm. Thus, we applied a Gaussian blur filter with a radius of 2 μm for the cross-sectional tissue images to reduce speckle noise.


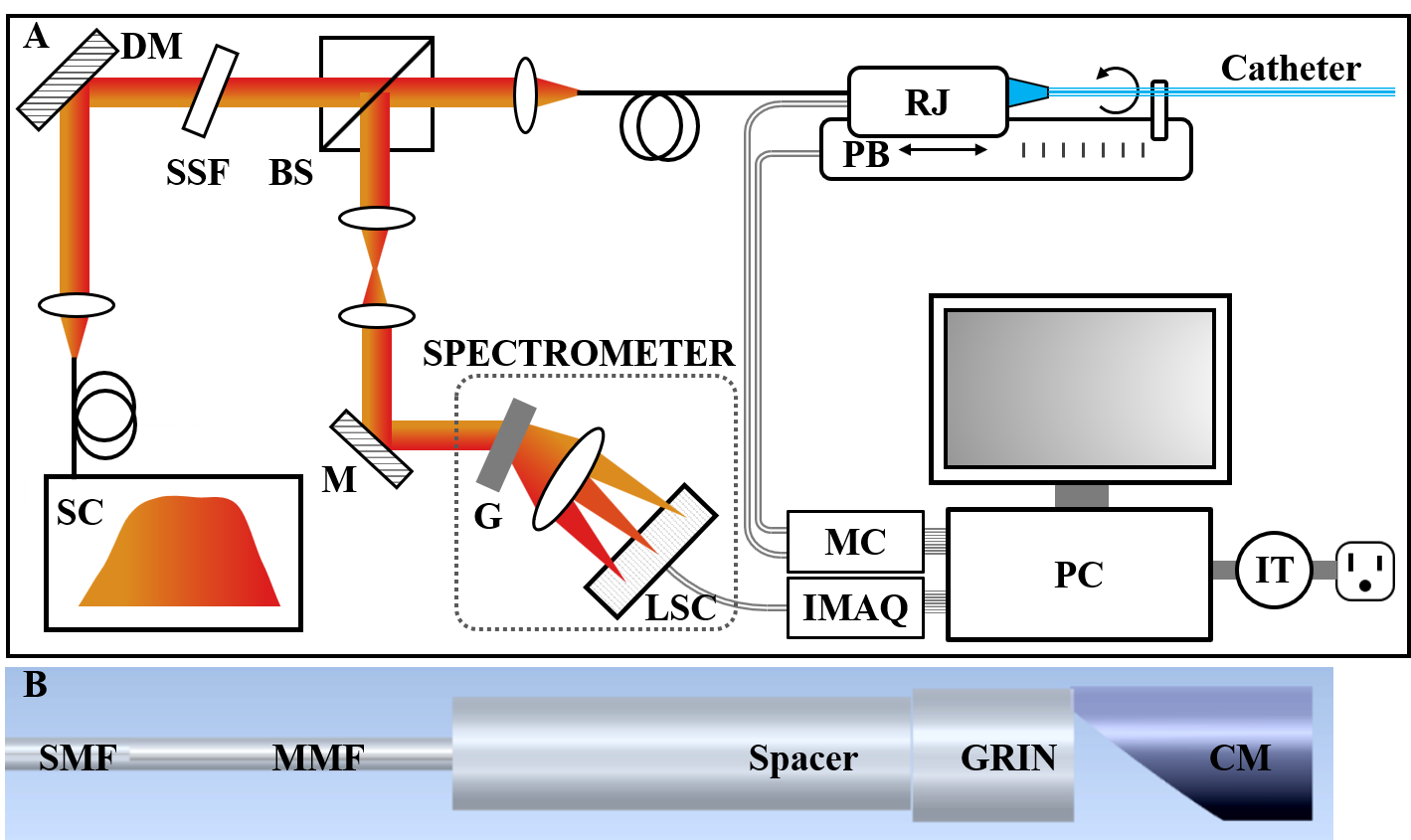


Figure S1. A. IVFMI imaging system comprising a broad-bandwidth low coherence interferometer, a rotary junction mounted onto a pullback stage, and a clinical-grade cardiovascular catheter containing a self-imaging wavefront division fiber optic probe^1^. SC: supercontinuum laser; DM: dichroic mirror; SSF: spectral shaping filter; BS: beam splitter; M: mirror; G: 1200 groves/mm grating; LSC: 8192-pixel line scan camera; RJ: rotary junction; PB: pullback stage; MC: motor controller; IMAQ: image acquisition board; PC: personal computer; IT: isolation transformer. B. A schematic of the catheter’s fiber probe consisting of a single-mode fiber, a segment of multimode fiber, a spacer, a GRIN lens, and a cylindrical mirror. SMF: single-mode fiber; MMF: multimode fiber; GRIN: graded-index lens; CM: cylindrical mirror.

1. **Resolution and depth of focus characterization**

The focused beam field varies subtly for different wavelengths, as shown in a simulation performed at the center wavelength (800 nm) and the blue (650 nm) and red (950 nm) ends of the operational spectra (Fig. S2A). The coaxially focused multimode (CAFM) beam consists of a Gaussian beam field generated by the fundamental mode, and multiple Bessel-like beam field generated by high order modes^2^. The intensity averaged beam profile suggests that a chromatic focal shift effect mitigates the field discontinuity caused by mode transition. According to the simulation, a 2-5-μm-diameter needle-like beam field can be maintained over approximately a 1.5 mm depth range that provides a significantly extended DOF compared to that of a conventional spatially coherent imaging system that only provides one Gaussian beam field with a much smaller DOF (<100 μm).

A cross-sectional image of a nanoparticle phantom obtained with the catheter is presented in Fig. S2B. As shown in Fig. S2C, the catheter has an average lateral resolution of 3-4 μm, starting from the outer surface of the sheath and extending to 1.5 mm away from the center of the catheter. And as shown in Fig. S2D, the axial resolution of the catheter is maintained at ~1.5 μm in tissue. Compared with the theoretical axial resolution (~1.4 μm), this minor axial resolution degradation could be in part due to spectral modification associated with chromatic focal shift effect, speckle, or inaccurate estimate of the medium’s refractive index.


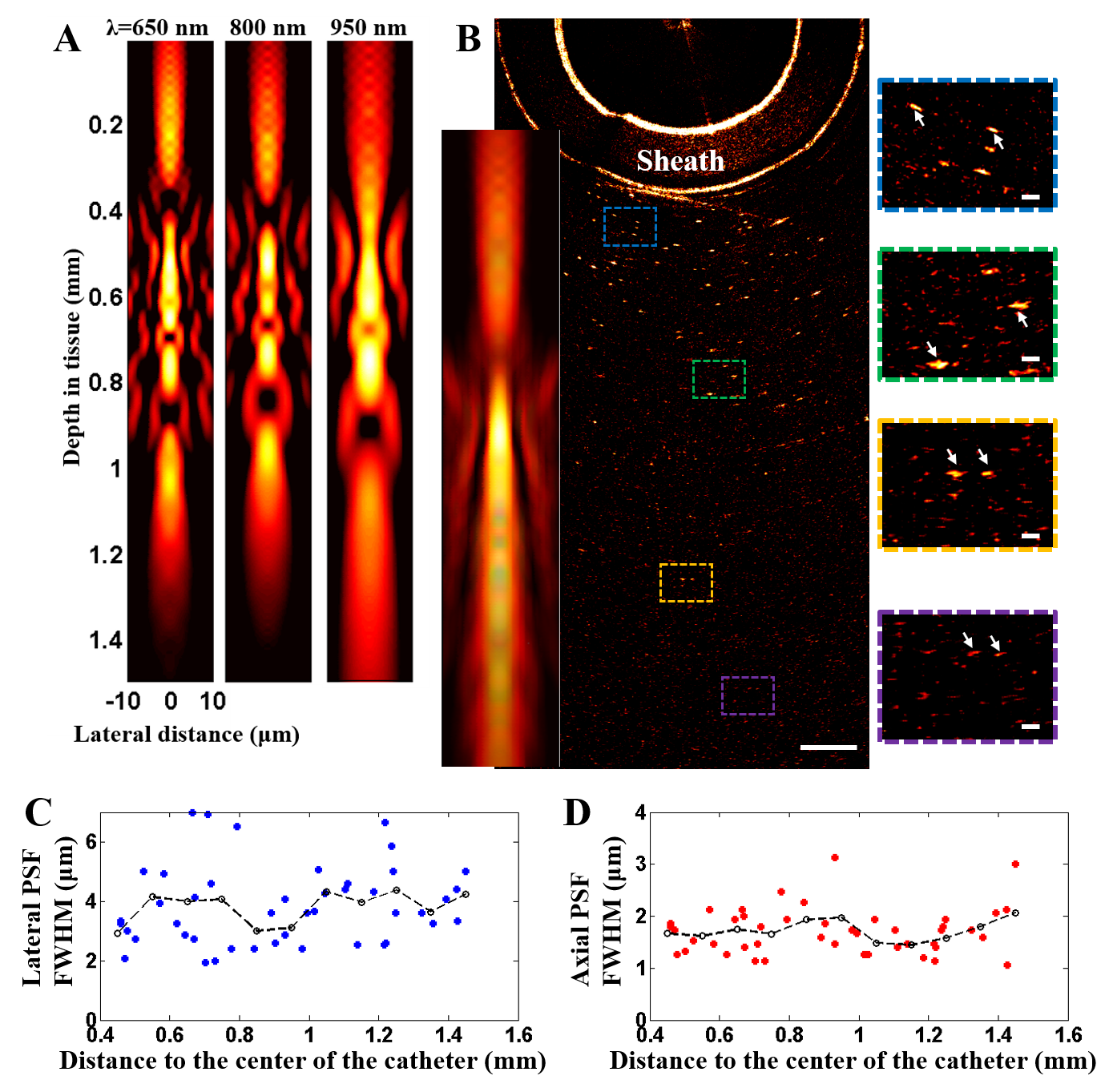


Figure S2. A. Simulated beam profiles in aqueous environment at wavelengths of 650 nm, 800 nm, and 950 nm. The beam profile is normalized by the peak intensity, and presented in dB scale, with a dynamic range of 16 dB. B. Cross-sectional image of a nanoparticle phantom overlaid with the simulated spectrally intensity-averaged beam profiles (spectrum starts at 650 nm and ends at 950 nm with a spectral resolution of 50 nm). The diameters of the nanoparticles are less than 1 μm. Four magnified images showing unaberrated PSFs over a broad depth range. White arrows indicate the nanoparticles. Scale bar in the main image: 100 μm; Scale bar in the magnified images: 10 μm. C. FWHM of the lateral PSF as a function of imaging depth; the black dot-circle line is the FWHM of the lateral PSF averaged every 100 μm. D. FWHM of the axial PSF along depth, and the black dot-circle line is the FWHM of the axial PSF averaged every 100 μm. A refractive index of 1.5 has been used to correct the optical path length in the medium.

1. **Pseudo-Bessel field side lobe analysis**

The beam profile in image space consists of a Gaussian beam field corresponding to the 0^th^ order mode and a pseudo-Bessel beam field corresponding to the high order modes. The transverse beam profiles for 800 nm wavelength were analyzed at three different depths. Figs. S3A, B, and C represent the transverse intensity distribution of the emitted beam field in log scale at these depths. Since the backscattered light from sample is collected by the same SMM fiber system that emits the light, the detected signal amplitude is proportional to the square of the transverse field amplitude distribution of the emitted beam profile. This confocal effect substantially reduces the intensity of the side lobe. When the confocal effect was considered, the peak of the side lobe was found to be more than 10 dB lower than the main lobe’s peak as shown in the table below (Tab. S1). Given that the dynamic range of the images are usually around 20-30 dB, a more than 10 dB intensity drop renders the side lobe artifact invisible. The phantom image shown in supplementary Fig. S2B also demonstrates this conclusion experimentally, where side lobe artifacts are not visualized in the nanoparticle point spread function.


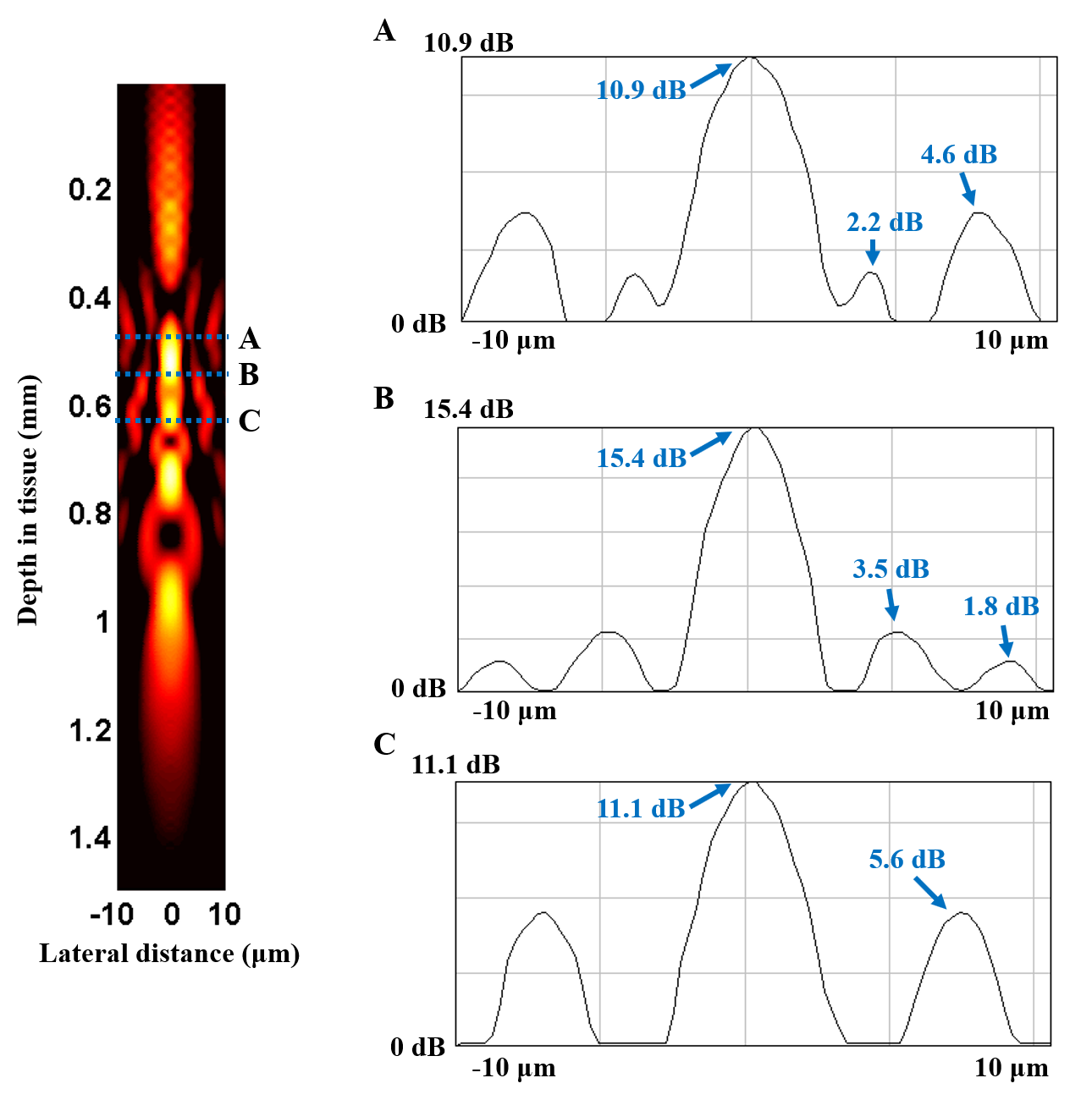


Figure S3. Side lobe analysis for the simulated beam profile at 800 nm wavelength. Transverse intensity distribution of the emitted field in log scale at three different depths are presented in A, B and C. The peak intensities of the main lobe and the side lobes are estimated. Note that the peak intensities presented in the plot are in the image space and do not take into account confocal detection by the core of the single-mode fiber.


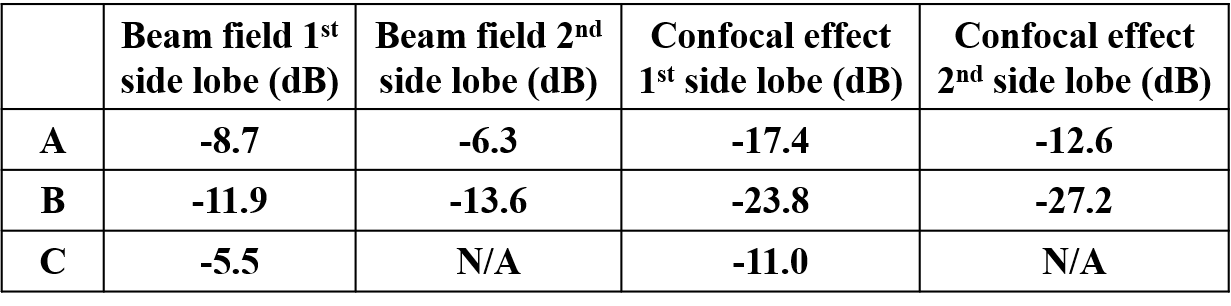


Table S1. Intensity difference between main lobe and side lobes at depths A, B, and C. Due to the confocal effect, the side lobes are observed to have intensities that are more than 10 dB lower than the main lobe. The 2^nd^ side lobe for depth C is not observed within the +/-10 μm range, thus, the entries are N/A for 2^nd^ side lobe of depth C.

1. **Tissue image speckle analysis**

A spatial frequency analysis was conducted to estimate how speckle correlates with resolution in tissue. The speckle pattern in Fig. S4A was Fourier-transformed and presented in log scale (Fig. S4B). A nonlinear least-square fit was applied to recover the modulation transfer function (MTF) based on a Gaussian model (Fig. S4C), where we observe a nearly-isotropic 2-dimensional MTF that has a Gaussian deviation approximates 0.2 cycles/μm (0.16 cycles/μm in lateral direction and 0.22 cycles/μm in axial direction) that translates to a 5 μm spatial separation, which is close to the size of the spatial filter applied to the image (a Gaussian blur filter of a radius of 2 μm).


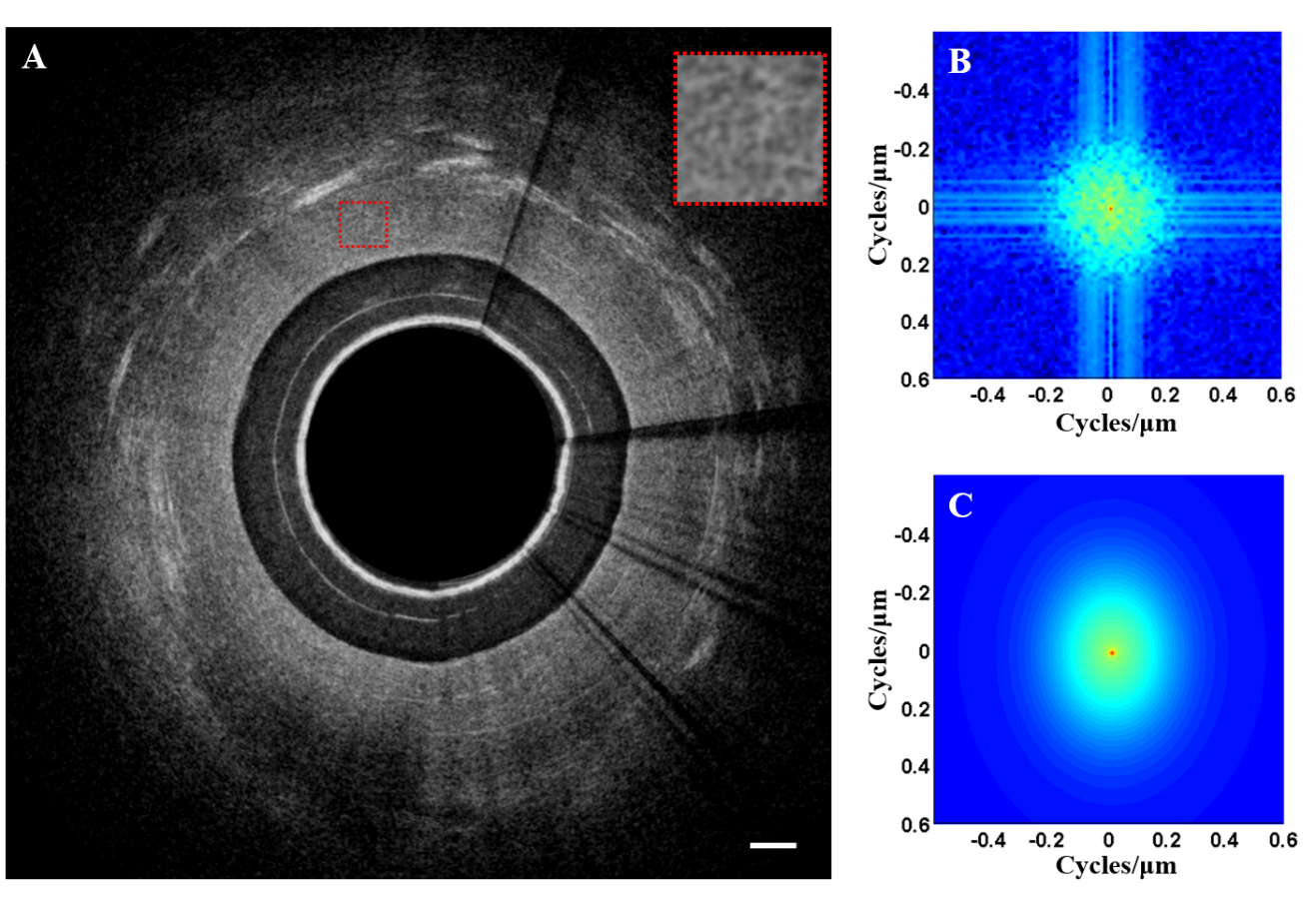


Figure S4. Tissue image speckle analysis. A. A cross-sectional image of human cadaver coronary artery acquired by IVFMI. The highlighted region is the region for speckle analysis. B. Fourier-transform of the speckle pattern in log scale. C. Estimated modulation transfer function obtained by the nonlinear least-square fit of B based on a Gaussian model, which shows a Gaussian deviation of approximately 0.2 cycle/μm. Scale bar: 100 μm.

References:

1. Biwei Yin, Kengyeh K. Chu, Chia-Pin Liang, Kanwarpal Singh, Rohith Reddy, and Guillermo J. Tearney, “μOCT imaging using depth of focus extension by self-imaging wavefront division in a common-path fiber optic probe,” Optics Express, **24**(5), 5555-5564 (2016).
2. Biwei Yin, Chulho Hyun, Joseph A. Gardecki, and Guillermo J. Tearney, “Extended depth of focus for coherence-based cellular imaging,” Optica, **4**(8), 959-965 (2017).
